# Advances and Future Perspective on Detection Technology of Human Norovirus

**DOI:** 10.3390/pathogens10111383

**Published:** 2021-10-26

**Authors:** Nan Wang, Guiying Pan, Ping Liu, Shaofeng Rong, Zhiyong Gao, Qianqian Li

**Affiliations:** 1 Department of Bioengineering, Shanghai Institute of Technology, Shanghai 201418, China; wn10010616@163.com (N.W.); 196071117@mail.sit.edu.cn (G.P.); pingliu1982@163.com (P.L.); rongshaofeng@163.com (S.R.); 2 Beijing Research Center for Preventive Medicine, Beijing Center for Disease Prevention and Control, Beijing 100013, China; zhiyonggao1@163.com

**Keywords:** human noroviruses, food-borne pathogens, detection technology, immunological methods, molecular detection, biosensor

## Abstract

Human norovirus (HuNoV) is a food-borne pathogen that causes acute gastroenteritis in people of all ages worldwide. However, no approved vaccines and antiviral drugs are available at present. Therefore, the development of accurate and rapid detection technologies is important in controlling the outbreak of HuNoVs. This paper reviewed the research progress on HuNoV detection, including immunological methods, molecular detection and biosensor technology. Immunological methods and molecular detection technologies are still widely used for HuNoV detection. Furthermore, biosensors will become an emerging developmental direction for the rapid detection of HuNoVs because of their high sensitivity, low cost, easy operation and suitability for onsite detection.

## 1. Introduction

Norovirus (NoV) is a single-stranded sense nonenveloped RNA virus, which belongs to the Caliciviridae family. It is classified into 10 genogroups (GI-GX) and further divided into 48 confirmed capsid genotypes on the basis of the amino acid sequences of the major capsid protein (VP1). Amongst the genogroups, GI, GII, GIV, GVIII and GX can infect humans, and they are commonly referred to as human noroviruses (HuNoVs) [1]. HuNoVs are recognised as the main food-borne pathogen causing acute gastroenteritis in humans worldwide. The proportion of food-borne infections caused by HuNoV in the world is 18% each year [2]. Statistics show that the global annual direct economic loss caused by HuNoVs is USD 4.2 billion, and the socioeconomic loss is USD 60.3 billion (of which, infants and children under 5 years old cost USD 39.8 billion) [3]. HuNoVs have become a leading factor in nonbacterial gastroenteritis [4,5,6].

**Figure 1:**
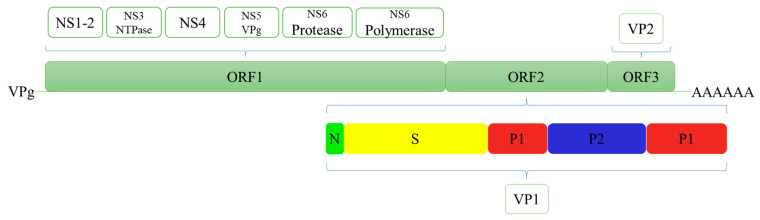
Structural and nonstructural proteins of Human norovirus.

The genome of HuNoV is divided into three open reading frames (ORFs) (Figure 1) [7]. ORF1 encodes six nonstructural proteins [8,9]. ORF2 and ORF3 encode the major capsid protein (VP1) and minor capsid protein (VP2), respectively. The VP1 is composed of a conserved S domain and a P domain [10]. The P domain is further divided into the P1 subdomain and a highly variable P2 subdomain. It has multiple neutralising epitopes, and it can bind to the known viral ligand, namely, histo-blood group antigens (HBGAs) [11]. The VP2 is located inside the capsid [12]. Therefore, VP1 or partial sequences of ORF1 are usually selected as the target sites for HuNoV detection.

HuNoV is highly infectious. It is usually transmitted through faecal–oral routes, aerosols, contaminated water and food, or direct human-to-human contact [13,14]. Various fresh foods such as berries, leafy vegetables and shellfish are carriers of HuNoVs [15,16,17]. However, HuNoVs detection in food and environmental samples are difficult due to the complicated matrix of backgrounds. Moreover, the high stability of virions against normal disinfection methods in these samples cause numerous food-borne HuNoV infections [18,19,20,21].

Another obstacle to developing efficient detection methods is the lack of mature HuNoV culture modes in vitro and in small animal models. Jones et al. reported the successful replication of HuNoV in human B cells. However, only HuNoV GII.4 can be replicated in this system [22,23]. Ettayebi et al. successfully developed a culture system using human intestinal enteroids. However, the HuNoV titres in this culture system are limited [24,25]. Animal models have also been explored. Dycke et al. reported HuNoV replication in zebrafish larvae. However, the highest virus titre has reached only 10^7^ PFU/mL [26]. Therefore, the inability to collect sufficient HuNoV makes studying various detection methods and developing antiviral strategies difficult.

At present, the control for HuNoV outbreaks primarily depends on the detection technology for achieving early detection, control and prevention. The most important goals for the development of HuNoV detection methods are high safety, good sensitivity, strong specificity, quickness and simplicity. With continuous research on the biological mechanism of HuNoVs, progress has been made in the detection of HuNoVs. According to different detection principles, HuNoV detection technologies can be divided into morphological methods based on HuNoV particles, immunological methods based on antigen–antibody reactions, molecular detection methods based on nucleic acids and biosensor methods which have developed in recent years. This article summarises the development of major detection technologies for HuNoVs.

## 2. Detection Techniques

### 2.1. Morphological Methods

In the early days (the 1970s–1980s), the clinical diagnosis methods for HuNoVs primarily included electron microscopy (EM) and immunoelectron microscopy (IEM) [27,28]. The limit of detection (LOD) for EM is high (>10^6^ virus particles/g sample). Thus, collecting samples (faeces or vomit) was necessary for testing during the acute phase of the disease [29]. Considering that the morphological characteristics of HuNoVs were not evident under the electron microscope, observation of viral morphological characteristics had strong subjectivity. Therefore, the detection rates and accuracy were low. IEM used the convalescent patients’ serum as antibodies to specifically capture HuNoV particles before identification. This method significantly improved the accuracy and sensitivity compared with EM. Although IEM was a sensitive and specific serological technology, its high technical requirements, the need to match the serum and the expensive equipment hindered its application in clinical testing.

### 2.2. Immunological Methods

Early immunological methods were based on the specific reaction between patients’ serum and HuNoV particles, for example, immune-adhesive haemagglutination assay (IAHA), radioimmunoassay (RIA), biotin-avidin immunoassay (BAI) and enzyme immunoassay (EIA) [30,31,32,33]. The IAHA can be used to assess the level of Norwalk virus antibodies in serum, but it cannot detect viral particles in clinical samples. The RIA used radioisotopes (I^125^) to label antibodies (anti-NoV IgG). However, the shelf life of labelled antibodies was short (less than 2 weeks), and the detection was time-consuming (approximately 6 days). It also had disadvantages such as high cost and a certain degree of risk. Compared with RIA, the shelf lives of labelled antibodies for BAI and EIA were longer, which was 3 months (−20 °C) and 6 months (4 °C), respectively. In brief, these methods primarily relied on a limited number of serum and clinical stool samples from infected volunteers. Moreover, the major shortcomings, such as long processing time, difficult matching of serum and limited detection sensitivity for clinical samples, hindered the development of immunological detection technology.

The Norwalk virus genome was successfully cloned until 1992. The VP1 was further expressed by the baculovirus system. The VP1 could self-assemble to form virus-like particles, which had the same immunogenicity as NoV. It provided an excellent antigen and antibody production platform and served as an alternative model to study the interaction between receptors from potential host cells and NoVs [34,35]. Since then, monoclonal antibodies (MAbs) and polyclonal antibodies (PAbs) against VLPs have been produced and widely used in the immunological detection of HuNoVs. Studies showed that hyperimmune serum obtained from VLP-immunised animals (mice, guinea pigs and rabbits) contributed high specificity and sensitivity to RIA, EIA and enzyme-linked immunosorbent assay (ELISA) detection methods compared with human serum samples [34,36]. In 1995, Herrmann et al. prepared MAbs against the NoV prototype 8FIIa strain. It was applied in EIA to detect NoV in stool samples [37]. The LOD was 1 ng/mL. The specificity was strong, and cross reaction was not detected. In 2007, Okame et al. used anti-GII.3 VLP PAbs as capture antibodies and three anti-GII.4 VLP MAbs as detection antibodies to conduct a sandwich ELISA. A total of eight genotypes (GI and GII) of HuNoVs from clinical samples were successfully detected [38]. In 2008, Takanashi et al. developed an immunochromatographic (ICG) test strip for the rapid detection of GI and GII HuNoVs in clinical stool samples [39]. Xu et al. developed a colloidal gold ICG assay for the detection of HuNoVs [40]. This system used the recombinant S protein of HuNoVs as the immunogen to prepare MAbs (capture antibodies and labelled antibodies) for the rapid (15 min) detection of GI and GII in clinical samples. Compared with RT-qPCR, the detection rate, specificity and consistency were 84.2%, 100.0% and 87.7%, respectively. A total of 10 genotypes of HuNoVs could be detected simultaneously. In addition, alkaline lysis was performed on HuNoV particles during pretreatment. This process could obtain several viral particles, and it is safe for people. Moreover, this method was easy to operate without the extraction of viral nucleic acids. Therefore, it is suitable for use as a point-of-care test or for preliminary screening of HuNoVs.

Commercial kits for the detection of HuNoVs based on immunological methods were developed. EIA-based kits mainly include IDEIA Norovirus (Oxoid Ltd., Hampshire, United Kingdom; two generations available) and RIDASCREEN Norovirus (R-Biopharm, Darmstadt, Germany; three generations available). ICG-based kits mainly include NOROTOP^®^ (ALL.DIAG SA, Strasbourg, France), ImmunoCardSTAT!^®^ Norovirus (Meridian Bioscience Europe, Nice, France), Ridaquick Norovirus (R-Biopharm, Darmstadt, Germany), and SD Bioline Norovirus (Standard Diagnostics, Inc., Kyonggi-do, Korea). Both commercial EIA- and ICG-based kits were evaluated for clinical sample testing [41,42,43,44,45,46,47]. The ICG-based kits have a shorter detection time (about 15–30 min) compared to EIA-based kits. Given the large antigenic differences and infectious ability amongst different genotypes of HuNoVs, the viral loads in foods and environmental samples were low. The viral loads in clinical samples were relatively high, but large differences were observed in different genotypes. Therefore, immunological methods such as ELISA and immune colloidal gold cannot satisfy the strict testing requirements because of the low sensitivity [48,49]. However, immunological methods have easy and fast operation and simple equipment requirements. As important techniques, immunological methods should be further improved. Immunological methods can be used for early screening of HuNoVs infection and for efficient diagnosis of disease in combination with molecular detection methods.

### 2.3. Molecular Detection Methods

The HuNoV molecular detection technology was primarily used to detect the conserved fragments of the NoV genome. The junction region of ORF1-ORF2 was mostly conserved in the NoV genome, which was widely used as a target for molecular detection [50,51]. To date, detection technologies based on HuNoV nucleic acid were primarily divided into two types according to different nucleic acid amplification methods. One is based on isothermal amplification, including reverse transcription-loop-mediated isothermal amplification (RT-LAMP), nuclear acid sequence-based amplification (NASBA) and recombinase polymerase amplification (RPA); the other was based on thermal cycling and amplification, including reverse transcription polymerase chain reaction(RT-PCR), quantitative real-time polymerase chain reaction (RT-qPCR), in situ capture-RT-qPCR (ISC-RT-qPCR) and reverse transcription digital polymerase chain reaction (RT-dPCR).

#### 2.3.1. Isothermal Amplification

The LAMP could be used to detect different subtypes of HuNoVs [52]. The LOD was approximately 10^3^ genomic copies/reaction tube. No cross reaction was observed amongst the subtypes. Based on this method, hydroxy naphthol blue dye was introduced into RT-LAMP to evaluate the results directly [53]. The sensitivity reached 10^3^ genomic copies per reaction tube, and the coincidence rate was 94.83% compared with RT-PCR detection. Then, a one-step RT-LAMP was developed. The sensitivity was 10 times higher than that of traditional RT-qPCR [54]. The RT-LAMP had high sensitivity, low cost and easy operation, which was suitable for onsite rapid detection. Moreover, multiplex RT-LAMP can promote this technology.

NASBA technology could be applied to detect NoVs. Greene et al. first reported that the LOD was 10^4^ RT-PCR-detectable units of NoVs RNA in a stool filtrate, and the detection time was 4–6 h [55]. Moore et al. detected GI and GII HuNoVs in clinical stool samples [56]. Compared with RT-PCR, the sensitivity and specificity of NASBA were 100% and 80%, respectively. Lamhoujeb et al. reported a real-time molecular beacon NASBA to detect GII HuNoVs in stool samples, which was 88.5% consistent with the RT-qPCR results [57]. NASBA does not require high-quality templates, and this technology has low mismatch rates, easy operation and high specificity and sensitivity. It is suitable for the rapid preliminary determination of the cause of infection, which can provide early warning for the early control of HuNoV infection.

RT-RPA is a new type of rapid real-time detection technology for HuNoV. The detection can be completed within 30 min at 40 °C, and the LOD reached 3.40 ± 0.20 log genomic copies. Compared with the routine RT-qPCR assay, RT-RPA is not sensitive to inhibitors, and it can detect complex samples [58]. Moreover, given its short processing time, determining the cause of infection to control the spread of infection became easy.

#### 2.3.2. Thermal Cycling Amplification

RT-PCR is the most widely used molecular detection technique because of its strong specificity and high sensitivity. The first RT-PCR detection was developed for the relatively conserved RNA polymerase gene in ORF1 [59]. However, primers designed for RT-PCR could not meet the genetic diversity of HuNoVs. In addition, RT-PCR has been applied in the ORF1-ORF2 junction region for the HuNoV genome (the most conserved region). However, RT-PCR requires high-quality RNA templates, and it is a time-consuming process and has high sensitivity to RNA inhibitors. Therefore, RT-qPCR was explored. Given its high sensitivity and specificity, RT-qPCR is currently known as the standard for the detection of HuNoVs in food and environmental samples. Kageyama et al. reported the first set of primer probes for the detection of GI and GII HuNoVs and established relative RT-qPCR [60]. Moreover, Jothikumar et al. established one-step TaqMan probe RT-qPCR to detect GI and GII HuNoVs in shellfish and clinical samples [61]. The method took 90 min, and the sensitivity increased by 10–100 times compared with the traditional one-step RT-PCR. Liu et al. updated the primers and probes and designed dual RT-qPCR to detect GI and GII HuNoVs, simultaneously [62]. Compared with the previously reported RT-qPCR, dual RT-qPCR had higher specificity and sensitivity with a processing time of 40 min. This method not only ensured the reliability of the results but also improved efficiency during detection for a large quantity of samples.

Virions have potential infectivity. However, RT-PCR and RT-qPCR, based on the detection of viral RNA, cannot distinguish infectious virions, inactive virions, or free RNA. It may result in misjudgment of the infection. In addressing the problem, Gilpatrick et al. used antibodies (PAbs for VLPs) and magnetic beads to capture viral particles from stool samples [63]. This method could remove inhibitors effectively and improve the accuracy of the detection. However, the HuNoV genotypes that can be detected by this method were limited. Afterwards, detection methods based on magnetic bead HBGAs and magnetic bead PGM were successively reported to detect HuNoVs particles instead of free RNA [64,65,66]. Wang et al. developed ISC-RT-qPCR using HBGAs to capture viral particles [67]. This method could evaluate the inactivation effect of HuNoVs by disinfection methods such as heating, chlorine and UV [68]. It was also used to detect potentially infectious HuNoVs in environmental water samples [69], clinical samples and commercial oyster samples [70]. The results showed that compared with traditional RT-qPCR, this method had higher sensitivity, particularly for potentially infectious HuNoVs. ISC-RT-qPCR simplified the steps for virion concentration and viral RNA extraction and eliminated the effects of PCR inhibitors and free RNA of HuNoVs. Therefore, ISC-RT-qPCR had great advantages in food, environmental and clinical sample detection.

New PCR-based detection techniques have been reported in recent years. Batule et al. developed a HRPzyme-PCR colorimetric method for HuNoV detection [71]. This method connected HRPzyme onto PCR primers. When a substrate was added into a PCR product, the change in colour caused by the HRPzyme reaction was recorded by a microplate reader. For the detection of GI and GII HuNoV in oyster samples, the recovery rates ranged from 92% to 105% by this method. Moreover, RT-dPCR was developed and widely used, which was suitable for samples with complex compositions and low viral loads. In this method, microfluidization or microdroplets are adopted to disperse the diluted nucleic acid solution into hundreds or even millions of microreactors or droplets on a chip. Then, the number of nucleic acid templates in each microreactor or droplet is less than or equal to one. After PCR, a microreactor or droplet containing the nucleic acid template will have a fluorescence signal. On the contrary, microreactor or droplet without the template will have no signal. Finally, the concentration or copy number of the target can be calculated according to the Poisson distribution principle and the proportion of positive droplets. dPCR has low detection limits, high accuracy and is not affected by the complex matrix of the samples. However, RT-dPCR requires expensive instrument, which hindered its widespread application [72,73,74,75].

Commercial kits for the detection of HuNoVs based on RT-qPCR were developed [76,77]. At present, the US Food and Drug Administration (FDA) has permitted three commercial kits that can be used for GI/GII HuNoV detection, including FilmArray GI panel (BioFire Diagnostics, Salt Lake City, UT, USA), Luminex xTag GI pathogen panel (GPP; Luminex Corporation, Toronto, Canada) and Nanosphere Verigene enteric pathogen test (Nanosphere, Inc., Northbrook, IL, USA) [78]. Chhabra et al. evaluated the performance of three commercial kits including Biofire’s Gastrointestinal Panel (FilmArray, BioFire Diagnostics, Salt Lake City, UT, USA), Luminex xTAG^®^ Gastrointestinal Pathogen Panel (GPP), and the TaqMan Array Card (TAC) for the detection of HuNoVs (GI/GII) in stool samples [79]. The sensitivities of FilmArray, GPP, and TAC system for norovirus GI and GII detection were 87.8%, 78.0%, and 87.8%, respectively. Zhuo et al. evaluated the Luminex xTag GPP. The results indicated that this kit was not sensitive to GII.2 and GII.3 HuNoVs [80]. Therefore, commercial kits were insufficient for the detection of multiple HuNoV genotypes. Furthermore, applicable kits may need to be updated on the basis of the HuNoVs epidemic virus survey.

### 2.4. Biosensor

In recent years, biosensors have shown great potential for application in pathogen detection because of their timeliness, stability, low cost and the possibility of integrating and miniaturising them into point-of-care testing devices [81,82,83,84,85]. A biosensor is usually composed of a biometric recognition element, transducer and signal processing unit. The interaction between the analyte and biometric recognition element is converted into a quantifiable signal output by the transducer [86]. The biometric recognition element is the key to determining the specificity of the biosensor. The selection of the biometric recognition element depends on the characteristics of the analyte. It should have high affinity and stability for the analyte. It includes enzymes (such as HRP), antibodies, ligands, nucleic acids and phages (for pathogenic bacteria detection), molecularly imprinted polymers, affinity and cells. Amongst them, antibodies are most commonly used for virus detection [87]. The current biosensors are primarily divided into electrochemical sensors, optical sensors and piezoelectric sensors according to the signal transduction mode [88]. Biosensors have become a new focus in the field of HuNoV detection (Table 1). Considerable research focused on the development of fast, sensitive, portable and easy-to-operate biosensors for the detection of HuNoVs in food, environmental or clinical samples.

#### 2.4.1. Electrochemical Biosensor

The electrochemical biosensor is a widely used biosensor [102]. Antibodies are commonly used as the biological recognition element in electrochemical biosensor. However, mAbs have high specificity and cost, whereas pAbs are relatively cheap, but they have poor specificity. Therefore, aptamers, peptides and other proteins are considered as biological recognition elements based on factors such as good stability and specificity, easy production, low cost and easy modification. The fixation of biometric components is also important. The stability and exposure of binding sites of biometric components are considered.

**Figure 2:**
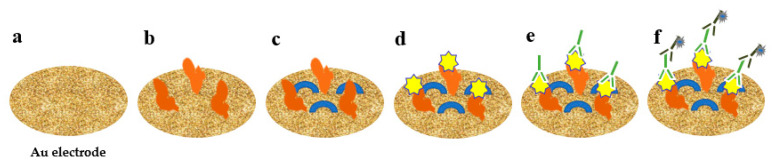
Detection of HuNoV using electrochemical biosensor with concanavalin-A. (**a**) The nanostructured gold electrode; (**b**) immobilized with ConA; (**c**) blocked by mercaptohexanol; (**d**) addition of HuNoV; (**e**) addition of detection antibody; (**f**) addition of secondary antibody.

**Figure 3:**
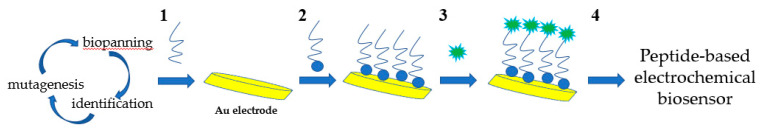
Schematic diagram of preparation of affinity peptide electrochemical sensor for norovirus detection. **1**, Selection of affinity peptide as molecular binders using phage display technique. **2**, Synthesis and immobilization of peptides. **3**, Addition of norovirus. **4**, Electrochemical detection.

**Figure 4:**
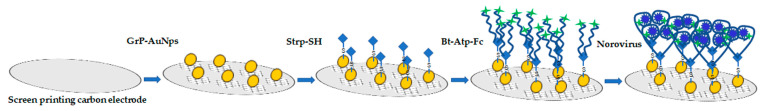
Schematic diagram of electrode functionalization and aptasensing for norovirus detection by screen-printed carbon electrodes. Grp–AuNPs: Graphene–gold nanoparticle composite, Strp-SH: Thiolated streptavidin, Bt-Atp-Fc: Biotin- and ferrocene-tagged aptamer.

Wang et al. developed an impedance immunosensor on the basis of interdigital array microelectrodes to detect avian influenza virus H5N1 [103]. Protein A was used to fix the Fc of the antibody on the electrode and to expose the Fab. It effectively improved virus capture efficiency. Hong et al. developed an electrochemical biosensor for HuNoV detection (it takes approximately 1 h) using concanavalin-A (ConA) as a biometric recognition element immobilised on a gold nanoelectrode (Figure 2) [89]. ConA had good sensitivity and selectivity, and the cost was only 2% of the cost for antibodies. This biosensor was used to detect HuNoVs in lettuce with a LOD of 60 genomic copies/mL. Wang et al. developed a miniature electrochemical biosensor on the basis of aptamer modification to detect HuNoV [90]. Murine norovirus (MNV) was used as an alternative model of HuNoVs. A thiolated AG3 aptamer was modified with FAM (specific binding to MNV) and fixed on a gold electrode to capture MNV. Then, cyclic voltammetry (CV) was used to characterise the fixation of the aptamer, and square wave voltammetry was used to characterise the MNV capture. This method was simple, sensitive and fast. Hwang et al. reported a highly sensitive and specific electrochemical biosensor for HuNoV detection based on sulfhydryl-modified affinity peptides immobilised on a gold electrode as a biorecognition element (Figure 3) [91]. The short-chain affinity peptides against the P2 domain of the recombinant VP1 (rP2) were screened as biorecognition elements by a phage display technology. Then, quartz crystal microbalance (QCM), CV and electrochemical impedance spectroscopy (EIS) were used for the detection of rP2 and HuNoVs GII.4. The LODs reached 99.8 nM and 7.8 genomic copies/mL, respectively. Chand and Neethirajan reported a microfluidic chip integrating screen-printed carbon electrodes and polydimethylsiloxane for the electrochemical detection of HuNoVs GII.4 VLPs (Figure 4) [92]. The chip was filled with silica magnetic beads to filter clinical samples and concentrate HuNoVs. The HuNoV-specific aptamers labelled with ferrocene (as redox probes) and biotin were immobilised on the carbon electrodes modified by graphene–gold nanoparticles and thiolated streptavidin. The HuNoVs were captured by the specific aptamer, which resulted in changes in the electrochemical signal. Then, differential pulse voltammetry analysis was used to determine and analyse the results. Using this method, the LOD for VLPs reached 100 pM. Baek et al. developed an impedance biosensor on the basis of NoroBP-nonFoul (Flexi)_2_ peptide-modified gold screen-printed electrode (SPE) for HuNoV GII.4 detection. In oyster samples, the LOD reached 1.7 genomic copies/mL with high sensitivity and good reproducibility [93].

To date, the most reported electrochemical biosensor detection methods use the classic three-electrode system (working electrode, counter electrode and reference electrode). Traditional solid electrodes are large, and they require complicated cleaning steps before use. However, SPE has low cost, less reagents, easy modification, flexible selection for solid-phase carriers (ceramics, paper, films, etc.) and inks (carbon, gold, etc.) and self-designed shape and size, which is suitable for mass production and portable or small devices [104,105]. Therefore, SPE has greater application advantages than traditional solid electrodes [105]. In addition, the combination of SPE and microfluidic technology can significantly reduce the consumption of reagents and samples. Multiple steps such as sample processing and detection can be integrated through a rational design to achieve a simple, fast and efficient testing [106,107,108,109].

The cost for the development of an electrochemical biosensor is important. The gold electrodes have strong electric signals, but they are expensive. On the contrary, carbon electrodes have a lower cost, but the electrical signals are weaker. Therefore, carbon electrodes are often modified by various methods. Gold nanoparticles (AuNPs) have good biocompatibility, excellent conductivity and high surface-to-volume ratio. AuNPs are commonly used modification materials, particularly for electrochemical immunosensors [110,111]. Lin et al. tested the signal amplification performance of electrochemical biosensors modified by AuNPs. The EIS results indicated that the signal was significantly enhanced [112]. Moreover, the shapes (nanoballs, nanorods, nanoislands, nanocages, etc.) and sizes of the gold nanomaterials can be controlled according to the needs [113].

#### 2.4.2. Optical Biosensor

The optical biosensors for virus detection primarily include surface-enhanced Raman spectroscopy (SERS) and surface plasmon resonance (SPR). SERS has been widely used in the detection of a variety of viruses, such as hepatitis B virus, influenza virus, adenovirus, rhinovirus, human immunodeficiency virus, respiratory syncytial virus and rotavirus [114]. However, no report has been found on the application of SERS in the detection and identification of HuNoV.

SPR biosensors have been widely applied in virus detection, such as avian influenza virus [115], Epstein–Barr virus [116], human hepatitis B virus [117] and Dengue virus [118]. Yakes et al. reported a quantitative SPR biosensor using feline calicivirus (FCV) as an alternative model of HuNoVs [94]. The results showed that FCV could be detected from purified cell lysates with a LOD of approximately 10^4^ TCID_50_ FCV/mL. Moreover, this biosensor had good renewability. Ashiba et al. developed an SPR-assisted quantum dot fluorescence biosensor with a V-shaped trench. For HuNoV VLP detection, the LOD reached 0.01 ng/mL [95]. Nasrin et al. reported an immunofluorescence nano-biosensor on the basis of localised SPR (LSPR) [96]. The linear range for detecting HuNoV VLPs was from 10^−14^ to 10^−9^ g/mL, and the LOD was 12.1 × 10^−15^ g/mL. For clinically isolated HuNoVs, the detection range was 10^2^–10^5^ genomic copies/mL, and the LOD was 95.0 genomic copies/mL. Furthermore, SPR can be used to analyse the binding kinetics and affinity of HuNoVs to ligands [119].

**Figure 5:**
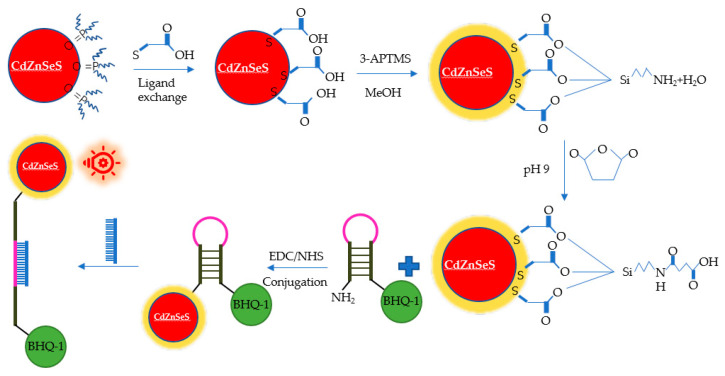
Schematic diagram of the alloyed QD-MB bioprobe for detection of norovirus RNA.

New optical detection technologies have been developed for HuNoV detection. Han et al. developed a 3D sliding paper-based analysis device. The results could be observed directly in 10 min without additional professional equipment [97]. HuNoV GII.4 in stool samples was detected, and the LOD was 9.5 × 10^4^ genomic copies/mL. Adegoke et al. developed an ultrasensitive SiO_2_-encapsulated alloyed CdZnSeS quantum dot-molecular beacon nano-biosensor for HuNoVs (Figure 5) [98]. The LODs of HuNoV RNA in human serum and buffer were 8.2 and 9.3 genomic copies/mL, respectively. Chung et al. reported a smartphone-based microfluidic paper analysis device [99]. The LODs of HuNoVs in deionised water and recycled wastewater were 1 genomic copy/μL and 10 genomic copies/μL, respectively. Ahmed et al. developed a colorimetric immunosensor based on graphene–gold nanoparticle (Grp–AuNPs) nanoprobes [100]. For HuNoV GII.4 VLP detection, the linear range was from 100 pg/mL to 10 μg/mL. The LOD was 92.7 pg/mL, which was 112 times lower than that of traditional ELISA. The sensitivity was 41 times higher than that of commercially available diagnostic kits. Lee et al. developed an ultra-sensitive sensor on the basis of a 3D total internal reflection scattering and defocusing microscope and a wavelength-dependent transmission grating to detect norovirus group-I capsid protein (NoVP). The LOD was 820 yoctomolar (yM, 10^−2^^4^ M), and the detection linear range was from 820 yM to 92.45 pM [101].

A piezoelectric biosensor, as a newly developed mass-based biosensor, can be used in virus detection. It measures small changes in mass caused by the binding of biomolecules (such as antibodies/antigens and enzymes/substrates) [120]. QCM is the most commonly used label-free piezoelectric biosensor [121]. It has been applied to virus detection, including avian influenza viruses [122,123,124] and hepatitis B virus [125,126]. Piezoelectric biosensors have ultrahigh sensitivity, and such biosensors are real-time, fast and quantifiable. However, improving their stability and anti-interference ability remains a challenge. Piezoelectric biosensors have shown potential applications, although few reports were focused on HuNoV detection using this technology.

## 3. Future Perspectives

Accurate and rapid detection technology is the main method to control the outbreak of HuNoVs before effective antiviral drugs or vaccines are developed. Development of good pretreatment methods for complex samples is the first important approach because it can significantly affect the accuracy and sensitivity of the detection method. At present, RT-qPCR is a reliable and common technology for detecting HuNoVs. The challenges are primarily focused on processing samples from different sources, distinguishing infectious and non-infectious viruses and the accurate assessment of the disease outbreaks. The immune colloidal gold test strip is a rapid detection method, which has simple operation and low cost. However, improving the detection sensitivity and extending the validity period are the keys to promoting this technology for further use. Biosensors are a promising detection technology. Future research must focus on finding highly selective and active biometric components and optimising fixation technology. Furthermore, good signal amplification methods should be explored to reduce the detection limit. In the future, methods for the optimization of the concentration of viral particles from food or environmental matrices need to be improved, because the viral contamination in these matrices is generally low. Meanwhile, the development of detection technology will focus on the improvement of the existing technology and exploration of new interdisciplinary methods.

## Figures and Tables

**Table 1:** Overview of biosensor assays for the detection of noroviruses.

Biosensor	Bioreceptor	Signal ^1^	Target	LOD (with Linear Range) ^2^	Reference
Electrochemistry	Concanavalin A	CV	NoV GII.4 subtype	35 genomic copies/mL (10^2^–10^6^ genomic copies/mL)	[89]
	MNV-specific aptamer	SWV	MHV-1 ^2^	(0–1.0 × 10^4^ PFU/mL)	[90]
	Noro-1 affinity peptide	QCM/CV/EIS	rP2/ NoV GII.4 subtype	rP2 (99.8 nM) GII.4 (7.8 genomic copies/mL)	[91]
	81-bases-long aptamer	DPV	GII VLPs	100 pM (100 pM–3.5 nM)	[92]
	NoroBP-nonFoul (FlexL)_2_ peptide	EIS	HuNoV GII.4 subtype	1.7 genomic copies/mL (0–10^5^ genomic copies/mL)	[93]
Optics	FCV antibody	SPR	FCV ^3^	≈10^4^ TCID_50_ FCV/mL	[94]
	Anti-HuNoV GII.4 monoclonal antibody (12A11)	SPR	GII.4 VLPs	0.01 ng/mL	[95]
	Anti-norovirus antibody (NS14)	LSPR	NoV GII VLPs/NoV	12.1 × 10^−15^ g/mL (10^−14^–10^−9^ genomic copies/mL) 95.0 genomic copies/mL (10^2^–10^5^ genomic copies/mL)	[96]
	Anti-norovirus GII.4 antibody (for capture) Anti-norovirus GII.4 capsid protein VP1 antibody (for detection)	The assay results could be visualised by the naked eye	NoV GII.4 subtype	9.5 × 10^4^ genomic copies/mL (1.58 × 10^5^–7.9 × 10^7^ genomic copies/mL)	[97]
	Molecular beacon probes (contained 20 bp complementary to NV genogroup II RNA)	UV–vis absorption and fluorescence emission measurements	NoV GII RNA (in human serum) NoV GII RNA (in buffer)	In human serum: 8.2 genomic copies/mL In buffer: 9.3 genomic copies/mL	[98]
	Anti-norovirus capsid protein VP1 polyclonal antibody	Benchtop fluorescence microscope	NoV GII (diluted in deionised water) NoV GII (diluted in reclaimed wastewater)	In deionised water: 1 genomic copies/μL In reclaimed wastewater: 10 genomic copies/μL	[99]
	Anti-norovirus antibody (NS14)	Colorimetric detection (OD_450 nm_)	HuNoV GII.4 VLP	92.7 pg/mL (100 pg/mL to 10 μg/mL)	[100]
	Monoclonal capture antibody (C01875M) Monoclonal detection antibody (C01874M)	3D Total internal reflection-scattering defocused imaging with wavelength-dependent transmission grating	NoV GI capsid protein VP1	820 yM (820 yM–92.45 pM)	[101]

^1^ CV, cyclic voltammetry; SWV, square wave voltammetry; QCM, quartz crystal microbalance; EIS, electrochemical impedance spectroscopy; DPV, differential pulse voltammetry; SPR, surface plasmon resonance; LSPR, localised SPR; UV, ultraviolet. ^2^ ng (nanogram, 10^−9^ g), µg (microgram, 10^−6^ g), pg (picogram, 10^−12^ g), yM (yoctomolar,10^−24^ M). ^3^ a surrogate of norovirus.

## Data Availability

The data presented in this study are contained within the article.

## References

[1] Chhabra P., Graaf M.D., Parra G.I., Chan C.W., Vinjé J. (2019). Updated classification of norovirus genogroups and genotypes. J. Gen. Virol..

[2] Ahmed S.M., Hall A.J., Robinson A.E., Verhoef L., Premkumar P., Parashar U.D., Koopmans M., Lopman B.A. (2014). Global prevalence of norovirus in cases of gastroenteritis: A systematic review and meta-analysis. Lancet Infect. Dis..

[3] Bartsch S.M., Lopman B.A., Sachiko O., Hall A.J., Lee B.Y., Olson D.R. (2016). Global economic burden of norovirus gastroenteritis. PLoS ONE.

[4] Hemming M., Räsänen S., Huhti L., Paloniemi M., Salminen M., Vesikari T. (2013). Major reduction of rotavirus, but not norovirus, gastroenteritis in children seen in hospital after the introduction of RotaTeq vaccine into the National Immunization Programme in Finland. Eur. J. Pediatr..

[5] Hallowell B.D., Parashar U.D., Curns A., Degroote N.P., Tate J.E. (2019). Trends in the laboratory detection of rotavirus before and after implementation of routine rotavirus vaccination-United States, 2000–2018. MMWR Morb. Mortal. Wkly. Rep..

[6] Jonesteller C.L., Burnett E., Yen C., Tate J.E., Parashar U.D. (2017). Effectiveness of rotavirus vaccination: A systematic review of the first decade of global postlicensure data, 2006–2016. Clin. Infect. Dis..

[7] Zheng D.P., Ando T., Fankhauser R.L., Beard R.S., Glass R.I., Monroe S.S. (2006). Norovirus classification and proposed strain nomenclature. Virology.

[8] Lee S., Liu H., Wilen C.B., Sychev Z.E., Desai C., Hykes B.L., Orchard R.C., McCune B.T., Kim K., Nice T.J. (2019). A secreted viral nonstructural protein determines intestinal norovirus pathogenesis. Cell Host Microbe.

[9] Jiang X., Wang M., Wang K., Estes M.K. (1993). Sequence and genomic organization of Norwalk virus. Virology.

[10] Prasad B.V., Hardy M.E., Dokland T., Bella J., Rossmann M.G., Estes M.K. (1999). X-ray crystallographic structure of the Norwalk virus capsid. Science.

[11] Ford-Siltz L.A., Tohma K., Parra G.I. (2021). Understanding the relationship between norovirus diversity and immunity. Gut Microbes.

[12] Vongpunsawad S., Venkataram Prasad B.V., Estes M.K. (2013). Norwalk virus minor capsid protein VP2 associates within the VP1 shell domain. J. Virol..

[13] Atmar R.L. (2010). Noroviruses: State of the art. Food Environ. Virol..

[14] Reymão T.K.A., Fumian T.M., Justino M.C.A., Hernandez J.M., Bandeira R.S., Lucena M.S.S., Teixeira D.M., Farias F.P., Silva L.D., Linhares A.C. (2018). Norovirus RNA in serum associated with increased fecal viral load in children: Detection, quantification and molecular analysis. PLoS ONE.

[15] Bozkurt H., Phan-Thien K.Y., Ogtrop F.V., Bell T., Mcconchie R. (2020). Outbreaks, occurrence, and control of norovirus and hepatitis a virus contamination in berries: A review. Crit. Rev. Food Sci. Nutr..

[16] Herman K.M., Hall A.J., Gould L.H. (2015). Outbreaks attributed to fresh leafy vegetables, United States, 1973–2012. Epidemiol. Infect..

[17] Gyawali P., Fletcher G.C., Mccoubery D.J., Hewitt J. (2019). Norovirus in shellfish: An overview of post-harvest treatments and their challenges. Food Control.

[18] Knight A., Haines J., Stals A., Li D., Uyttendaele M., Knight A., Jaykus L. (2016). A systematic review of human norovirus survival reveals a greater persistence of human norovirus RT-qPCR signals compared to those of cultivable surrogate viruses. Int. J. Food Microbiol..

[19] Becker B., Dabisch-Ruthe M., Pfannebecker J. (2020). Inactivation of murine norovirus on fruit and vegetable surfaces by vapor phase hydrogen peroxide. J. Food Prot..

[20] Barclay L., Park G.W., Vega E., Hall A., Parashar U., Vinjé J., Lopman B. (2014). Infection control for norovirus. Clin. Microbiol. Infect..

[21] Cook N., Knight A., Richards G.P. (2016). Persistence and elimination of human norovirus in food and on food contact surfaces: A critical review. J. Food Prot..

[22] Jones M.K., Watanabe M., Zhu S., Graves C.L., Keyes L.R., Grau K.R., Gonzalez-Hernandez M.B., Iovine N.M., Wobus C.E., Vinje J. (2014). Enteric bacteria promote human and mouse norovirus infection of B cells. Science.

[23] Jones M.K., Grau K.R., Costantini V., Kolawole A.O., De Graaf M., Freiden P., Graves C.L., Koopmans M., Wallet S.M., Tibbetts S.A. (2015). Human norovirus culture in B cells. Nat. Protoc..

[24] Ettayebi K., Crawford S.E., Murakami K., Broughman J.R., Karandikar U., Tenge V.R., Neill F.H., Blutt S.E., Zeng X., Qu L. (2016). Replication of human noroviruses in stem cell-derived human enteroids. Science.

[25] Zou W.Y., Blutt S.E., Crawford S.E., Ettayebi K., Zeng X., Saxena K., Ramani S., Karandikar U.C., Zachos N.C., Estes M.K. (2019). Human intestinal enteroids: New models to study gastrointestinal virus infections. Methods Mol. Biol..

[26] Dycke J.V., Ny A., Conceição-Neto N., Maes J., Hosmillo M., Cuvry A., Goodfellow I., Nogueira T.C., Verbeken E., Matthijnssens J. (2019). A robust human norovirus replication model in zebrafish larvae. PLoS Pathog..

[27] Thornhill T.S., Wyatt R.G., Kalica A.R., Dolin R., Chanock R.M., Kapikian A.Z. (1977). Detection by immune electron microscopy of 26-to 27-nm viruslike particles associated with two family outbreaks of gastroenteritis. J. Infect. Dis..

[28] Riepenhoff-Talty M., Barrett H.J., Spada B.A., Ogra P.L. (1983). Negative staining and immune electron microscopy as techniques for rapid diagnosis of viral agents. Ann. N. Y. Acad. Sci..

[29] Richards A.F., Lopman B., Gunn A., Curry A., Ellis D., Cotterill H., Ratcliffe S., Jenkins M., Appleton H., Gallimore C.I. (2003). Evaluation of a commercial ELISA for detecting Norwalk-like virus antigen in faeces. J. Clin. Virol..

[30] Kapikian A.Z., Greenberg H.B., Cline W.L., Kalica A.R., Wyatt R.G., James H.D., Lloyd N.L., Chanock R.M., Ryder R.W., Kim H.W. (1978). Prevalence of antibody to the Norwalk agent by a newly developed immune adherence hemagglutination assay. J. Med. Virol..

[31] Greenberg H.B., Wyatt R.G., Valdesuso J., Kalica A.R., London W.T., Chanock R.M., Kapikian A.Z. (1978). Solid-phase microtiter radioimmunoassay for detection of the Norwalk strain of acute nonbacterial, epidemic gastroenteritis virus and its antibodies. J. Med. Virol..

[32] Gary G.W., Kaplan J.E., Stine S.E., Anderson L.J. (1985). Detection of Norwalk virus antibodies and antigen with a biotin-avidin immunoassay. J. Clin. Microbiol..

[33] Herrmann J.E., Nowak N.A., Blacklow N.R. (1985). Detection of Norwalk virus in stools by enzyme immunoassay. J. Med. Virol..

[34] Jiang X., Wang M., Graham D.Y., Estes M.K. (1992). Expression, self-assembly, and antigenicity of the Norwalk virus capsid protein. J. Virol..

[35] Xi J.N., Graham D.Y., Wang K.N., Estes M.K. (1990). Norwalk virus genome cloning and characterization. Science.

[36] Green K.Y., Lew J.F., Jiang X., Kapikian A.Z., Estes M.K. (1993). Comparison of the reactivities of baculovirus-expressed recombinant Norwalk virus capsid antigen with those of the native Norwalk virus antigen in serologic assays and some epidemiologic observations. J. Clin. Microbiol..

[37] Herrmann J.E., Blacklow N.R., Matsui S.M., Lewis T.L., Estes M.K., Ball J.K., Brinker J.P. (1995). Monoclonal antibodies for detection of Norwalk virus antigen in stools. J. Clin. Microbiol..

[38] Okame M., Shiota T., Hansman G., Takagi M., Yagyu F., Takanashi S., Phan T.G., Shimizu Y., Kohno H., Okitsu S. (2007). Anti-norovirus polyclonal antibody and its potential for development of an antigen-ELISA. J. Med. Virol..

[39] Takanashi S., Okame M., Shiota T., Takagi M., Yagyu F., Tung P.G., Nishimura S., Katsumata N., Igarashi T., Okitsu S. (2008). Development of a rapid immunochromatographic test for noroviruses genogroups I and II. J. Virol. Methods.

[40] Xu M., Lu F., Lyu C., Wu Q., Zhang J., Tian P., Xue L., Xu T., Wang D. (2021). Broad-range and effective detection of human noroviruses by colloidal gold immunochromatographic assay based on the shell domain of the major capsid protein. BMC Microbiol..

[41] Khamrin P., Nguyen T.A., Phan T.G., Satou K., Masuoka Y., Okitsu S., Maneekarn N., Nishio O., Ushijima H. (2008). Evaluation of immunochromatography and commercial enzyme-linked immunosorbent assay for rapid detection of norovirus antigen in stool samples. J. Virol. Methods.

[42] Okitsu-Negishi S., Okame M., Shimizu Y., Phan T.G., Tomaru T., Kamijo S., Sato T., Yagyu F., Muller W.E.G., Ushijima H. (2006). Detection of norovirus antigens from recombinant virus-like particles and stool samples by a commercial norovirus enzyme-linked immunosorbent assay kit. J. Clin. Microbiol..

[43] Kirby A., Gurgel R.Q., Dove W., Vieira S.C.F., Cunliffe N.A., Cuevas L.E. (2010). An evaluation of the RIDASCREEN and IDEIA enzyme immunoassays and the RIDAQUICK immunochromatographic test for the detection of norovirus in faecal specimens. J. Clin. Virol..

[44] Kim H.S., Hyun J., Kim J.S., Song W., Kang H.J., Lee K.M. (2012). Evaluation of the SD Bioline Norovirus rapid immunochromatography test using fecal specimens from Korean gastroenteritis patients. J. Virol. Methods.

[45] Jonckheere S., Botteldoorn N., Vandecandelaere P., Frans J., Laffut W. (2017). Multicenter evaluation of the revised RIDA^®^ QUICK test (N1402) for rapid detection of norovirus in a diagnostic laboratory setting. Diagn. Microbiol. Infect. Dis..

[46] Bruin E.D., Duizer E., Vennema H., Koopmans M.P.G. (2006). Diagnosis of Norovirus outbreaks by commercial ELISA or RT-PCR. J. Virol. Methods.

[47] Ambert-Balay K., Pothier P. (2013). Evaluation of 4 immunochromatographic tests for rapid detection of norovirus in faecal samples. J. Clin. Virol..

[48] Bruggink L.D., Catton M.G., Marshall J.A. (2013). Evaluation of the Bioline Standard Diagnostics SD immunochromatographic norovirus detection kit using fecal specimens from Australian gastroenteritis incidents. Diagn. Microbiol. Infect. Dis..

[49] Gray J.J., Kohli E., Ruggeri F.M., Vennema H., Sánchez-Fauquier A., Schreier E., Gallimore C.I., Iturriza-Gomara M., Giraudon H., Pothier P. (2007). European multicenter evaluation of commercial enzyme immunoassays for detecting norovirus antigen in fecal samples. Clin. Vaccine Immunol..

[50] Katayama K., Shirato-Horikoshi H., Kojima S., Kageyama T., Oka T., Hoshino F.B., Fukushi S., Shinohara M., Uchida K., Suzuki Y. (2002). Phylogenetic analysis of the complete genome of 18 Norwalk-like viruses. Virology.

[51] Kojima S., Kageyama T., Fukushi S., Hoshino F.B., Shinohara M., Uchida K., Natori K., Takeda N., Katayama K. (2002). Genogroup-specific PCR primers for detection of Norwalk-like viruses. J. Virol. Methods.

[52] Fukuda S., Takao S., Kuwayama M., Shimazu Y., Miyazaki K. (2006). Rapid detection of norovirus from fecal specimens by real-time reverse transcription-loop-mediated isothermal amplification assay. J. Clin. Microbiol..

[53] Luo J., Xu Z., Nie K., Ding X., Guan L., Wang J., Xian Y., Wu X., Ma X. (2014). Visual detection of norovirus genogroup II by reverse transcription loop-mediated isothermal amplification with hydroxynaphthol blue dye. Food Environ. Virol..

[54] Jeon S.B., Seo D.J., Oh H., Kingsley D.H., Choi C. (2017). Development of one-step reverse transcription loop-mediated isothermal amplification for norovirus detection in oysters. Food Control.

[55] Greene S.R., Moe C.L., Jaykus L.A., Cronin M., Grosso L., Aarle P.V. (2003). Evaluation of the NucliSens Basic Kit assay for detection of Norwalk virus RNA in stool specimens. J. Virol. Methods.

[56] Moore C., Clark E.M., Gallimore C.I., Corden S.A., Westmoreland D. (2004). Evaluation of a broadly reactive nucleic acid sequence based amplification assay for the detection of noroviruses in faecal material. J. Clin. Virol..

[57] Lamhoujeb S., Charest H., Fliss I., Ngazoa S., Jean J. (2009). Real-time molecular beacon NASBA for rapid and sensitive detection of norovirus GII in clinical samples. Can. J. Microbiol..

[58] Moore M.D., Jaykus L.A. (2017). Development of a recombinase polymerase amplification assay for detection of epidemic human noroviruses. Sci. Rep..

[59] Vinjé J. (2015). Advances in laboratory methods for detection and typing of norovirus. J. Clin. Microbiol..

[60] Kageyama T., Kojima S., Shinohara M., Uchida K., Fukushi S., Hoshino F.B., Takeda N., Katayama K. (2003). Broadly reactive and highly sensitive assay for Norwalk-like viruses based on real-time quantitative reverse transcription-PCR. J. Clin. Microbiol..

[61] Jothikumar N., Lowther J.A., Henshilwood K., Lees D.N., Hill V.R., Vinje J. (2005). Rapid and sensitive detection of noroviruses by using TaqMan-based one-step reverse transcription-PCR assays and application to naturally contaminated shellfish samples. Appl. Environ. Microbiol..

[62] Liu D., Zhang Z., Wu Q., Tian P., Geng H., Xu T., Wang D. (2020). Redesigned duplex RT-qPCR for the detection of GI and GII human noroviruses. Engineering.

[63] Gilpatrick S.G., Schwab K.J., Estes M.K., Atmar R.L. (2000). Development of an immunomagnetic capture reverse transcription-PCR assay for the detection of Norwalk virus. J. Virol. Methods.

[64] Cannon J.L., Vinjé J. (2008). Histo-blood group antigen assay for detecting noroviruses in water. Appl. Environ. Microbiol..

[65] Tian P., Engelbrektson A., Mandrell R. (2008). Two-log increase in sensitivity for detection of norovirus in complex samples by concentration with porcine gastric mucin conjugated to magnetic beads. Appl. Environ. Microbiol..

[66] Dancho B.A., Chen H., Kingsley D.H. (2012). Discrimination between infectious and non-infectious human norovirus using porcine gastric mucin. Int. J. Food Microbiol..

[67] Wang D., Xu S., Yang D., Young G.M., Tian P. (2014). New in situ capture quantitative (real-time) reverse transcription-PCR method as an alternative approach for determining inactivation of Tulane Virus. Appl. Environ. Microbiol..

[68] Wang D., Tian P. (2014). Inactivation conditions for human norovirus measured by an in situ capture-qRT-PCR method. Int. J. Food Microbiol..

[69] Tian P., Yang D., Shan L., Li Q., Liu D., Wang D. (2018). Estimation of human norovirus infectivity from environmental water samples by in situ capture RT-qPCR method. Food Environ. Virol..

[70] Zhou Z., Tian Z., Li Q., Tian P., Wu Q., Wang D., Shi X. (2017). In situ capture RT-qPCR: A new simple and sensitive method to detect human norovirus in oysters. Front. Microbiol..

[71] Batule B.S., Kim S.U., Mun H., Choi C. (2018). Colorimetric detection of norovirus in oyster samples through DNAzyme as a signaling probe. J. Agric. Food Chem..

[72] Polo D., Schaeffer J., Fournet N., Le Saux J., Parnaudeau S., McLeod C., Le Guyader F.S. (2016). Digital PCR for quantifying norovirus in oysters implicated in outbreaks, France. Emerg. Infect. Dis..

[73] Monteiro S., Santos R. (2017). Nanofluidic digital PCR for the quantification of Norovirus for water quality assessment. PLoS ONE.

[74] Bartsch C., Hoeper D., Maede D., Johne R. (2018). Analysis of frozen strawberries involved in a large norovirus gastroenteritis outbreak using next generation sequencing and digital PCR. Food Microbiol..

[75] Coudray-Meunier C., Fraisse A., Martin-Latil S., Guillier L., Delannoy S., Fach P., Perelle S. (2015). A comparative study of digital RT-PCR and RT-qPCR for quantification of Hepatitis A virus and Norovirus in lettuce and water samples. Int. J. Food Microbiol..

[76] Suffredini E., Pepe T., Ventrone I., Croci L. (2011). Norovirus detection in shellfish using two Real-Time RT-PCR methods. New Microbiol..

[77] Navidad J.F., Griswold D.J., Gradus M.S., Bhattacharyya S. (2013). Evaluation of Luminex xTAG gastrointestinal pathogen analyte-specific reagents for high-throughput, simultaneous detection of bacteria, viruses, and parasites of clinical and public health importance. J. Clin. Microbiol..

[78] Binnicker M. (2015). Multiplex molecular panels for diagnosis of gastrointestinal infection: Performance, result interpretation, and cost-effectiveness. J. Clin. Microbiol..

[79] Chhabra P., Gregoricus N., Weinberg G.A., Halasa N., Chappell J., Hassan F., Selvarangan R., Mijatovic-Rustempasic S., Ward M.L., Bowen M. (2017). Comparison of three multiplex gastrointestinal platforms for the detection of gastroenteritis viruses. J. Clin. Virol..

[80] Zhuo R., Cho J., Qiu Y.Y., Parsons B.D., Lee B.E., Chui L., Freedman S.B., Pang X. (2017). High genetic variability of norovirus leads to diagnostic test challenges. J. Clin. Virol..

[81] Clark L.C., Lyons C. (1962). Electrode systems for continuous monitoring in cardiovascular surgery. Ann. N. Y. Acad. Sci..

[82] Montagnana M., Caputo M., Giavarina D., Lippi G. (2009). Overview on self-monitoring of blood glucose. Clin. Chim. Acta.

[83] Xiang Y., Lu Y. (2011). Using personal glucose meters and functional DNA sensors to quantify a variety of analytical targets. Nat. Chem..

[84] Lan T., Zhang J., Lu Y. (2016). Transforming the blood glucose meter into a general healthcare meter for in vitro diagnostics in mobile health. Biotechnol. Adv..

[85] Zeng L., Gong J., Rong P., Liu C., Chen J. (2019). A portable and quantitative biosensor for cadmium detection using glucometer as the point-of-use device. Talanta.

[86] Vo-Dinh T., Cullum B. (2000). Biosensors and biochips: Advances in biological and medical diagnostics. Fresenius J. Anal. Chem..

[87] Justino C.I.L., Freitas A.C., Pereira R., Duarte A.C., Santos T.A.P.R. (2015). Recent developments in recognition elements for chemical sensors and biosensors. TrAC Trend. Anal. Chem..

[88] Saylan Y., Erdem Z., Nal S., Denizli A. (2019). An alternative medical diagnosis method: Biosensors for virus detection. Biosensors.

[89] Hong S.A., Kwon J., Kim D., Yang S. (2015). A rapid, sensitive and selective electrochemical biosensor with concanavalin A for the preemptive detection of norovirus. Biosens. Bioelectron..

[90] Wang N., Kitajima M., Mani K., Kanhere E., Whittle A.J., Triantafyllou M.S., Miao J. Miniaturized Electrochemical Sensor Modified with Aptamers for Rapid Norovirus Detection. Proceedings of the 2016 IEEE 11th Annual International Conference on Nano/Micro Engineered and Molecular Systems (NEMS 2016).

[91] Hwang H.J., Ryu M.Y., Park C.Y., Ahn J., Park H.G., Choi C., Ha S., Park T.J., Park J.P. (2017). High sensitive and selective electrochemical biosensor: Label-free detection of human norovirus using affinity peptide as molecular binder. Biosens. Bioelectron..

[92] Chand R., Neethirajan S. (2017). Microfluidic platform integrated with graphene-gold nano-composite aptasensor for one-step detection of norovirus. Biosens. Bioelectron..

[93] Baek S.H., Kim M.W., Park C.Y., Choi C., Kailasa S.K., Park J.P., Park T.J. (2019). Development of a rapid and sensitive electrochemical biosensor for detection of human norovirus via novel specific binding peptides. Biosens. Bioelectron..

[94] Yakes B.J., Papafragkou E., Conrad S.M., Neill J.D., Ridpath J.F., Burkhardt W., Kulka M., Degrasse S.L. (2013). Surface plasmon resonance biosensor for detection of feline calicivirus, a surrogate for norovirus. Int. J. Food Microbiol..

[95] Ashiba H., Sugiyama Y., Wang X., Shirato H., Higo-Moriguchi K., Taniguchi K., Ohki Y., Fujimaki M. (2017). Detection of norovirus virus-like particles using a surface plasmon resonance-assisted fluoroimmunosensor optimized for quantum dot fluorescent labels. Biosens. Bioelectron..

[96] Fahmida N., Chowdhury A.D., Takemura K., Lee J., Adegoke O., Deo V.K., Abe F., Suzuki T., Park E.Y. (2018). Single-step detection of norovirus tuning localized surface plasmon resonance-induced optical signal between gold nanoparticles and quantum dots. Biosens. Bioelectron..

[97] Han K.N., Choi J., Kwon J. (2016). Three-dimensional paper-based slip device for one-step point-of-care testing. Sci. Rep..

[98] Adegoke O., Seo M., Kato T., Kawahito S., Park E.Y. (2016). An ultrasensitive SiO_2_-encapsulated alloyed CdZnSeS quantum dot-molecular beacon nanobiosensor for norovirus. Biosens. Bioelectron..

[99] Chung S., Breshears L.E., Perea S., Morrison C.M., Betancourt W.Q., Reynolds K.A., Yoon J.Y. (2019). Smartphone-based paper microfluidic particulometry of norovirus from environmental water samples at the single copy level. ACS Omega.

[100] Ahmed S.R., Takemeura K., Li T., Kitamoto N., Tanaka T., Suzuki T., Park E.Y. (2017). Size-controlled preparation of peroxidase-like graphene-gold nanoparticle hybrids for the visible detection of norovirus-like particles. Biosens. Bioelectron..

[101] Lee S., Ahn S., Chakkarapani S.K., Kang S.H. (2019). Supersensitive detection of the norovirus immunoplasmon by 3D total internal reflection scattering defocus microscopy with wavelength-dependent transmission grating. ACS Sens..

[102] Da Silva E.T.S.G., Souto D.E.P., Barragan J.T.C., Giarola J.d.F., de Moraes A.C.M., Kubota L.T. (2017). Electrochemical biosensors in point-of-care devices: Recent advances and future trends. ChemElectroChem.

[103] Wang R., Wang Y., Lassiter K., Li Y., Hargis B., Tung S., Berghman L., Bottje W. (2009). Interdigitated array microelectrode based impedance immunosensor for detection of avian influenza virus H5N1. Talanta.

[104] Taleat Z., Khoshroo A., Mazloum-Ardakani M. (2014). Screen-printed electrodes for biosensing: A review (2008–2013). Microchim. Acta.

[105] Alonso-Lomillo M.A., Domínguez-Renedo O., Arcos-Martínez M.J. (2010). Screen-printed biosensors in microbiology; a review. Talanta.

[106] Smith S., Madzivhandila P., Ntuli L., Bezuidenhout P., Zheng H., Land K. (2019). Printed paper–based electrochemical sensors for low-cost point-of-need applications. Electrocatalysis.

[107] Shi H., Nie K., Dong B., Long M., Xu H., Liu Z. (2019). Recent progress of microfluidic reactors for biomedical applications. Chem. Eng. J..

[108] Sackmann E.K., Fulton A.L., Beebe D.J. (2014). The present and future role of microfluidics in biomedical research. Nature.

[109] Coltro W.K.T., Cheng C., Carrilho E., de Jesus D.P. (2014). Recent advances in low-cost microfluidic platforms for diagnostic applications. Electrophoresis.

[110] Jans H., Huo Q. (2012). Gold nanoparticle-enabled biological and chemical detection and analysis. Chem. Soc. Rev..

[111] Guo S., Wang E. (2007). Synthesis and electrochemical applications of gold nanoparticles. Anal. Chim. Acta.

[112] Lin C.C., Chen L.C., Huang C.H., Ding S.J., Chang C.C., Chang H.C. (2008). Development of the multi-functionalized gold nanoparticles with electrochemical-based immunoassay for protein A detection. J. Electroanal. Chem..

[113] Xiao T., Huang J., Wang D., Meng T., Yang X. (2020). Au and Au-Based nanomaterials: Synthesis and recent progress in electrochemical sensor applications. Talanta.

[114] Ambartsumyan O., Gribanyov D., Kukushkin V., Kopylov A., Zavyalova E. (2020). SERS-Based Biosensors for Virus Determination with Oligonucleotides as Recognition Elements. Int. J. Mol. Sci..

[115] Bai H., Wang R., Hargis B., Lu H., Li Y. (2012). A SPR aptasensor for detection of avian influenza virus H5N1. Sensors.

[116] Riedel T., Rodriguez-Emmenegger C., Andres D.L.S.P., Bědajánková A., Jinoch P., Boltovets P.M., Brynda E. (2014). Diagnosis of Epstein-Barr virus infection in clinical serum samples by an SPR biosensor assay. Biosens. Bioelectron..

[117] Choi Y., Lee G., Ko H., Chang Y.W., Kang M., Pyun J. (2014). Development of SPR biosensor for the detection of human hepatitis B virus using plasma-treated parylene-N film. Biosens. Bioelectron..

[118] Omar N.A.S., Fen Y.W., Abdullah J., Sadrolhosseini A.R., Kamil Y.M., Fauzi N.I.M., Hashim H.S., Mahdi M.A. (2020). Quantitative and selective surface plasmon resonance response based on a reduced graphene oxide–polyamidoamine nanocomposite for detection of dengue virus E-proteins. Nanomaterials.

[119] Shang J., Piskarev V.E., Xia M., Huang P., Jiang X., Likhosherstov L.M., Novikova O.S., Newburg D.S., Ratner D.M. (2013). Identifying human milk glycans that inhibit norovirus binding using surface plasmon resonance. Glycobiology.

[120] Miroslav P. (2018). Overview of piezoelectric biosensors, immunosensors and DNA sensors and their applications. Materials.

[121] Saylan Y., Akgnüllü S., Yavuz H., Ünal S., Denizli A. (2019). Molecularly imprinted polymer based sensors for medical applications. Sensors.

[122] Li D., Wang J., Wang R., Li Y., Abi-Ghanem D., Berghman L., Hargis B., Lu H. (2011). A nanobeads amplified QCM immunosensor for the detection of avian influenza virus H5N1. Biosens. Bioelectron..

[123] Wang R., Li Y. (2013). Hydrogel based QCM aptasensor for detection of avian influenza virus. Biosens. Bioelectron..

[124] Wang R., Wang L., Callaway Z.T., Lu H., Huang T.J., Li Y. (2017). A nanowell-based QCM aptasensor for rapid and sensitive detection of avian influenza virus. Sens. Actuators B Chem..

[125] Yao C., Zhu T., Tang J., Wu R., Chen Q., Chen M., Zhang B., Huang J., Fu W. (2008). Hybridization assay of hepatitis B virus by QCM peptide nucleic acid biosensor. Biosens. Bioelectron..

[126] Giamblanco N., Conoci S., Russo D., Marletta G. (2015). Single-step label-free hepatitis B virus detection by a piezoelectric biosensor. RSC Adv..

